# Patient perspectives on humeral lengthening in achondroplasia: association between treatment timing and acceptance

**DOI:** 10.1007/s00590-026-04804-z

**Published:** 2026-06-23

**Authors:** Cristina Giuli, Osvaldo Palmacci, Camilla Ravaioli, Raffaele Vitiello, Giulio Maccauro, Maria Beatrice Bocchi

**Affiliations:** 1 Catholic University of the Sacred Heart, Rome, Italy; 2 Agostino Gemelli University Polyclinic, Rome, Italy

**Keywords:** Achondroplasia, Humeral lengthening, Patient reported outcome measures, FGFR3, Short stature

## Abstract

**Purpose:**

Achondroplasia is associated with upper-limb shortening, which may impair independence in daily activities. While lower-limb lengthening is commonly performed, humeral lengthening is often delayed. This study evaluated the functional impact of upper-limb shortening and patient perspectives regarding humeral lengthening.

**Methods:**

A single-centre cross-sectional questionnaire study was conducted in 27 patients with achondroplasia divided into three groups according to treatment status. Outcomes included independence in daily activities, functional and aesthetic satisfaction, and willingness to undergo humeral lengthening.

**Results:**

Twenty-four patients completed the survey (response rate: 89%). Patients who underwent humeral lengthening reported improved daily function and high satisfaction (4/5, 80%). Among untreated adults, 2 out of 5 (2/5, 40%) expressed regret, with persistent difficulties. In pediatric patients, satisfaction was generally low, whereas 9 out of 14 (9/14, 64%) expressed interest in future surgery.

**Conclusion:**

Humeral lengthening was associated with improved function and satisfaction. Earlier integration may warrant consideration in future treatment protocols.

**Level of evidence:**

IV.

**Supplementary Information:**

The online version contains supplementary material available at 10.1007/s00590-026-04804-z.

## Introduction

Achondroplasia is the most common skeletal dysplasia and is characterized by disproportionate short stature with rhizomelic limb shortening [1, 2]. It occurs in approximately 4.6 per 100,000 live births and is caused by activating variants in the fibroblast growth factor receptor-3 (FGFR3) gene, resulting in impaired endochondral ossification [3, 4].

Although medical therapies are evolving, orthopedic surgery, particularly limb-lengthening procedures, continues to play a central role in improving autonomy and function [4- 6]. Achieving a “functional height,” typically referring to final adult height after limb lengthening, has been proposed as a target to improve independence in activities of daily living, although no universally accepted threshold exists [7]. However, upper-limb rhizomelia may further contribute to limitations in daily activities and personal care [8-10].

Compared with other etiologies, patients with achondroplasia typically tolerate greater lengthening, demonstrate faster healing, and show lower complication rates [11]. Multiple lower-limb lengthening protocols combining different devices and techniques have been described, all reporting satisfactory outcomes; therefore, protocol selection often depends on surgeon preference [12–15,19].

Despite this, humeral lengthening is typically performed at the end of the overall treatment pathway, even though it is a well-tolerated procedure that allows patients to maintain independence throughout distraction and consolidation [2, 5, 13, 14]. Isolated lower-limb lengthening may lead to unfavorable limb proportions, potentially worsening functional limitations such as dressing and self-care difficulties [9].

Although the initiation of lower-limb lengthening has recently shifted to earlier ages (7–11 years) [15], humeral lengthening is still commonly performed during late adolescence, after years of demanding treatment. As a result, upper-limb lengthening in achondroplasia remains relatively uncommon and underrepresented in the literature.

Given the observed lower adherence to the humeral phase of the treatment pathway, a better understanding of patient perspectives regarding treatment sequencing and shared decision-making is needed.

This study aimed to explore patient perspectives on humeral lengthening in achondroplasia across different stages of the limb-lengthening pathway by addressing the following questions:

1. How does upper-limb shortening affect daily activities in patients with achondroplasia?
2. How do patients perceive the functional and aesthetic value of humeral lengthening?
3. Is the timing of humeral lengthening within the treatment pathway associated with patient acceptance of the procedure?

## Materials and methods

### Patients

Patients with achondroplasia followed at our pediatric orthopaedic and rare diseases outpatient clinic between 2020 and 2023 were considered eligible for this cross-sectional study.

A structured survey was developed by the clinician responsible for the care of all participants and distributed to patients and/or their families after institutional approval. The questionnaire was sent to all 27 patients in March 2024, and data collection was completed in May 2024.

The study was conducted in accordance with the Declaration of Helsinki and was approved by the Institutional Review Board of our Orthopedic and Traumatology Institute (February 2024). Written informed consent for the scientific use of clinical data was obtained according to institutional protocol.

All patients in active follow-up who voluntarily agreed to participate were included. Exclusion criteria were non-achondroplastic diagnosis, refusal to participate, incomplete or unfinished questionnaires, and late submissions.

After review of clinical records, patients were assigned to three groups by two researchers (O.P. and M.B.B.):

- Group A: patients who underwent bilateral humeral lengthening simultaneously using a monolateral external fixator
- Group B: adult patients who had not undergone humeral lengthening
- Group C: pediatric patients currently undergoing lower-limb lengthening

Group C was analyzed separately, as it represents a pediatric population still undergoing treatment and therefore not directly comparable to adult cohorts.

Returned questionnaires were screened by two additional researchers (C.G. and C.R.), and those with substantial missing data or inconsistencies were excluded. In case of disagreement regarding completeness or eligibility, a senior consultant (R.V.) was consulted to reach a final consensus. Only complete and reliable responses were included in the final analysis. No missing responses were identified in questionnaires included in the final dataset.

Demographic and clinical characteristics of the study population are summarized in Table 1.

**Table 1:** Demographics and clinical characteristics of study population

Variable	Group A	Group B	Group C
No. of patients	5	5	14
Sex (M/F)	4/1	4/1	7/7
Mean age at the time of filling in the questionnaire	23.6 ± 11.3 years	33.8 ± 11.2 years	13.8 ± 1.5 years
Period of lower-limb lengthening	2015–2023	1994–early 2000s	2020–2023
Mean age at initiation of lower-limb lengthening	8–9 years	10–11 years	8–9 years
Mean age at completion of lower-limb lengthening	16.8 ± 2.5 years	20 ± 0.8 years	ongoing
Mean age at the time of bilateral humeral lengthening	16.8 ± 2.5 years	Not performed	Not performed yet
Mean number of lengthening episodes	5	4	2
Typical treatment sequence	Tibia–Femur–Tibia–Femur–Humerus	Tibia–Femur–Tibia–Femur–Humerus	Tibia–Femur–Tibia–Femur–Humerus
Mean duration in external fixation	6–8 months per phase	10–12 months per phase	6–8 months per phase

### Methods

A structured questionnaire was developed to assess patient attitudes toward bilateral humeral lengthening and overall adherence to the limb-lengthening pathway in individuals with achondroplasia.

The questionnaire was designed based on domains and constructs previously explored in the achondroplasia and upper-limb literature [3, 8]. Items addressing satisfaction with humeral lengthening, perceived functional improvement, and willingness to undergo the procedure were adapted from previously published surveys on humeral lengthening in achondroplasia [16, 17]. Questions regarding independence in activities of daily living and perceived upper-limb function were developed based on domains commonly explored in validated upper-extremity assessment tools, including QuickDASH and PODCI [18, 19].

Although QuickDASH assesses upper-extremity function, PODCI evaluates broader pediatric functional outcomes, and disease-specific PROMs for achondroplasia evaluate psychosocial and functional aspects of achondroplasia, none of these tools specifically evaluate upper-limb lengthening, treatment sequencing, adherence to the limb-lengthening pathway, or patient attitudes toward humeral lengthening. Therefore, an ad hoc exploratory questionnaire was developed for the specific aims of this study.

Prior to administration, the questionnaire was reviewed in a preliminary pilot group to assess clarity and comprehensibility, with particular attention to age-appropriate wording for pediatric participants. Separate questionnaire versions were developed for adult and pediatric participants using simplified and age-appropriate wording. Pediatric patients completed the questionnaire with parental assistance when necessary; however, parents were instructed to support comprehension of the questions without influencing responses.

All questionnaires included self-reported items addressing:

- Demographic characteristics
- Physical function
- Overall functional outcome
- Aesthetic perception
- Acceptance of humeral lengthening

The questionnaire was administered via email in March 2024. Participants were given a response window of four weeks to complete and return the survey. No remuneration or incentives were provided for participation.

### Outcome measures

#### Physical function

Patients rated their independence in performing common upper-limb activities of daily living, including dressing, combing/washing hair, putting on shoes, performing intimate hygiene, and placing their hands into trouser pockets. Responses were recorded as dichotomous variables (“yes/no”). Dichotomous responses were selected to simplify questionnaire completion across different age groups and to facilitate interpretation in this exploratory study.

Patients in Group A evaluated these abilities both before and after humeral lengthening.

#### Functional outcome

Overall functional satisfaction was assessed using a 5-point Likert scale, reflecting patients’ perceived ability to use their upper limbs in daily life. Group A completed both pre- and post-operative evaluations.

#### Aesthetic perception

Patients rated the aesthetic appearance of their upper limbs using a 5-point Likert scale. Group A completed both pre- and post-lengthening assessments.

#### Acceptance and association with treatment timing

To explore the association between treatment timing and acceptance of humeral lengthening, each group responded to a specific statement:

- Group A: “I would choose to undergo humeral lengthening again.”
- Group B: “If I could go back, I would have humeral lengthening surgery.”
- Group C: “In the future, I would like to undergo humeral lengthening.”

Responses were recorded using a 5-point Likert scale.

Patients in Group B were also asked to report the reasons for not undergoing humeral lengthening.

#### Additional measures

Across all groups, adherence to the lower-limb lengthening pathway (yes/no) and the perceived importance of lower- and upper-limb lengthening were assessed using a 10-point scale (1 = not relevant, 10 = essential for activities of daily living).

Participants were also given the opportunity to provide additional free-text comments.

### Statistical analysis

Descriptive statistics were used to summarize patient characteristics and questionnaire responses. Continuous variables are presented as means and standard deviations, while categorical variables are reported as frequencies and percentages. Confidence intervals for proportions were calculated using the Wilson method.

Given the exploratory design of the study and the limited sample size, no inferential statistical analyses were performed, as the study was not powered to detect statistically significant differences between groups.

All analyses were performed using Microsoft Excel (Microsoft Corp., Redmond, WA, USA).

## Results

A total of 24 patients completed the survey.

### Impact of upper-limb shortening on daily activities in patients with achondroplasia

Independence in activities of daily living (ADLs) varied across groups.

In Group A, all patients (5/5) reported improvement across the assessed activities of daily living after humeral lengthening.

In Group B, patients were independent in most tasks; however, 3 out of 5 (3/5, 60%) reported difficulties with intimate hygiene and placing their hands into trouser pockets.

In Group C, functional limitations were more evident: 6 out of 14 (6/14, 43%) of patients reported difficulties with intimate hygiene and 7 out of 14 (7/14, 50%) with placing their hands into trouser pockets.

The negative impact of upper-limb shortening on daily activities is summarized in Table 2.

**Table 2:** Negative impact of upper-limb shortening on activities of daily living

Activity	Group A (Pre-op)	Group A (Post-op)	Group B	Group C
Hair care	1/5 (20%)	0/5 (0%)	0/5 (0%)	0/14 (0%)
Putting on shoes	2/5 (40%)	0/5 (0%)	0/5 (0%)	0/14 (0%)
Intimate hygiene	3/5 (60%)	0/5 (0%)	3/5 (60%)	6/14 (43%)
Hands in pockets	4/5 (80%)	0/5 (0%)	3/5 (60%)	7/14 (50%)
Dressing	1/5 (20%)	0/5 (0%)	0/5 (0%)	0/14 (0%)

Values indicate the number and percentage of patients reporting difficulty in performing each activity independently

### Patient perception of the functional and aesthetic value of humeral lengthening

Patients in Group A reported substantial functional improvement after humeral lengthening, with 3 out of 5 patients (3/5, 60%) totally agreeing and 2 out of 5 (2/5, 40%) agreeing that the procedure enhanced upper-limb function.

Aesthetic satisfaction improved markedly, shifting from predominantly low preoperative satisfaction scores to high postoperative satisfaction.

Overall satisfaction with the procedure was high. Four out of five patients in Group A (4/5, 80%) stated that they would undergo humeral lengthening again (Table 3). The only patient who would not repeat the procedure had experienced a complication, specifically delayed bone union, which was treated with curettage, autologous bone grafting, and intramedullary fixation after removal of the external fixator.

**Table 3:** Functional satisfaction, aesthetic perception, and acceptance of humeral lengthening

Outcome	Group A	Group B	Group C
Aesthetic satisfaction before humeral lengthening	0/5 satisfied (0%); Likert: all rated 2	1/5 satisfied (20%); Likert: 2 × 2, 1 × 3, 2 × 4	4/14 satisfied (28.5%); Likert: 1 × 1, 6 × 2, 4 × 3, 3 × 4
Functional satisfaction before humeral lengthening	0/5 satisfied (0%); Likert: all rated 2	3/5 satisfied (60%); Likert: 2 × 4, 3 × 3	1/14 satisfied (7%); Likert: 2 × 2, 8 × 3, 4 × 4
Importance of lower limb-lengthening (1–10)	9.4 ± 1.2	9 ± 0.8	9.5 ± 0.8
Importance of humeral lengthening (1–10)	9.4 ± 0.8	7.3 ± 2	7.6 ± 3.2
Willingness to undergo humeral lengthening	4/5 (80%; 95% CI, 37.6–96.4%)^a^	2/5 (40%; 95% CI, 11.8–76.9%)	9/14 (64%; ^a^95% CI, 38.8–83.7%)

Satisfaction was assessed using a 5-point Likert scale (1 = completely dissatisfied, 5 = completely satisfied)

^a^Confidence intervals for proportions were calculated using the Wilson method

In contrast, Group B showed more heterogeneous perceptions. While 3 out of 5 patients (3/5, 60%) were satisfied with functional performance, only 1 out of 5 (1/5, 20%) were satisfied with the appearance of their upper limbs. Two patients (2/5, 40%) regretted not undergoing humeral lengthening, two (2/5, 40%) expressed neutrality, and one (1/5, 20%) remained uninterested in the procedure.

In Group C, satisfaction was generally low: 4 out of 14 patients (4/14, 28.5%) were satisfied with upper-limb aesthetics, and only 1 out of 14 (1/14, 7%) with upper-limb function. Additionally, 9 out of 14 patients (9/14, 64%) expressed interest in undergoing humeral lengthening in the future.

### Association between treatment timing and acceptance of humeral lengthening

Differences in treatment sequencing appeared to be associated with differences in acceptance of humeral lengthening.

In Group A, all patients had completed lower-limb lengthening before humeral surgery, at a mean age of 16.8 years. They rated the importance of upper- and lower-limb lengthening equally (9.4/10), suggesting that undergoing lower-limb procedures did not reduce their interest in humeral lengthening.

In Group B, 3 out of 5 patients (3/5, 60%) declined humeral lengthening due to the prolonged and demanding nature of the lower-limb lengthening process, whereas 2 out of 5 (2/5, 40%) cited limited perceived benefit.

The perceived importance of lower-limb lengthening was higher than that of humeral lengthening (9/10 vs 7.3/10).

In Group C, despite ongoing lower-limb treatment, pediatric patients reported interest in future humeral lengthening (9/14, 64%) and rated its importance at 7.6/10.

A limited number of participants provided free-text comments. Untreated patients reported lack of interest due to perceived limited benefit or age, whereas treated patients described improved quality of life. Patients awaiting treatment expressed expectations of improved daily function and greater social integration.

Functional satisfaction, aesthetic perception, and acceptance of humeral lengthening are summarized in Table 3.

## Discussion

While both lower- and upper-limb lengthening in achondroplasia have been associated with functional and psychosocial benefits, the upper-limb phase remains the least frequently performed step in the limb-lengthening pathway [14, 16, 17, 20].

In this study, we explored patient perspectives on humeral lengthening, focusing on satisfaction, reasons for non-adherence, and the role of treatment timing.

Patients who underwent humeral lengthening reported improvement in activities of daily living together with increased aesthetic satisfaction, consistent with previous reports describing functional benefit and high patient satisfaction after humeral lengthening [14, 16, 17]. In untreated patients, intimate hygiene represented the area of greatest dependence, whereas dissatisfaction with upper-limb aesthetics was common. These findings are consistent with previous studies describing the impact of rhizomelic upper-limb shortening on daily function [7, 8].

Adult patients who discontinued the lengthening pathway frequently cited the prolonged and demanding nature of lower-limb lengthening as the main reason for refusing humeral lengthening. Older age at completion of lower-limb lengthening may also have contributed to reduced willingness to pursue additional procedures, as Group B patients completed treatment later than Group A (20 ± 0.8 years vs 16.8 ± 2.5 years). In addition, historical differences in treatment protocols may have influenced patient attitudes. Earlier cohorts underwent longer and more demanding external-fixation-based protocols, whereas more recent lower-limb lengthening strategies may reduce treatment burden and improve tolerability. In contrast, humeral lengthening still commonly relies on external fixation and is generally performed after years of cumulative treatment. This cumulative burden may partly explain the lower adherence to humeral lengthening observed in some patients.

The complication profile of humeral lengthening should also be considered. In our cohort, one patient experienced delayed bone union requiring additional surgery. Previous studies similarly reported complications including fractures, shoulder instability, pin-site infections, premature consolidation, and nerve-related complications [14, 16, 17, 21]. Therefore, although humeral lengthening may provide meaningful functional benefits, careful patient selection and counseling remain essential.

Finally, emerging pharmacological therapies such as vosoritide may progressively influence future treatment strategies in achondroplasia [24, 25]. Although these therapies may reduce the number of patients seeking extensive lower-limb lengthening, their long-term impact on upper-limb function and future indications for upper-limb lengthening remains uncertain.

### Limitations

This study has several limitations. First, the questionnaire was not validated and may therefore have introduced measurement bias, although it was developed using domains derived from previously published surveys and standardized upper-extremity assessment tools to address the specific aims of this exploratory study [16–19]. Second, the small sample size and single-center design limit generalizability and may have introduced selection bias. Differences between groups may also reflect variations in treatment era and historical limb-lengthening protocols, therefore, Groups A and B should not be considered directly comparable cohorts. In addition, Group A retrospectively evaluated preoperative function after surgery, introducing potential recall bias. Finally, no inferential statistical analyses were performed due to the exploratory design and limited sample size; therefore, findings should be interpreted descriptively. Despite these limitations, this study provides useful insights into patient perspectives on humeral lengthening and may contribute to a better understanding of patient attitudes and shared decision-making.

## Conclusion

This survey provides insight into patient perspectives on humeral lengthening in achondroplasia at different stages of the limb-lengthening pathway. Upper-limb shortening negatively affected both daily activities and aesthetic perception, while humeral lengthening was associated with improved function and satisfaction in treated patients. However, the cumulative burden of prolonged treatment may reduce willingness to undergo additional procedures. These findings highlight the importance of considering patient attitudes, expectations, and treatment burden during shared decision-making. Earlier integration of humeral lengthening may warrant consideration in future treatment protocols.

## Supplementary Information

Below is the link to the electronic supplementary material.

Supplementary Material 1

Supplementary Material 2

Supplementary Material 3

## Data Availability

De-identified datasets generated and/or analyzed during the current study are available from the corresponding author upon reasonable request.

## Abbreviations

ADLs: Activities of daily living
FGFR3: Fibroblast growth factor receptor-3

## References

[1] Kopits SE (1976) Orthopedic complications of dwarfism. Clin Orthop Relat Res 114:153–179 816586

[2] Pauli RM (2019) Achondroplasia: a comprehensive clinical review. Orphanet J Rare Dis 14:1. 10.1186/s13023-018-0972-6 30606190 10.1186/s13023-018-0972-6 PMC6318916

[3] Cattaneo R, Villa A, Catagni M, Tentori L (1988) Limb lengthening in achondroplasia by Ilizarov’s method. Int Orthop 12(3):173–179. 10.1007/BF00547160 3182120 10.1007/BF00547160

[4] Vaidya SV, Song HR, Lee SH, Suh SW, Keny SM, Telang SS (2006) Bifocal tibial corrective osteotomy with lengthening in achondroplasia: an analysis of results and complications. J Pediatr Orthop 26(6):788–793. 10.1097/01.bpo.0000242429.83866.97 17065948 10.1097/01.bpo.0000242429.83866.97

[5] Kim SJ, Pierce W, Sabharwal S (2014) The etiology of short stature affects the clinical outcome of lower limb lengthening using external fixation: a systematic review of 18 trials involving 547 patients. Acta Orthop 85(2):181–186. 10.3109/17453674.2014.899856 24650027 10.3109/17453674.2014.899856 PMC3967262

[6] Batibay SG, Balci HI, Bayram S, Chodza M, Goksoy S, Hurmeydan OM et al (2020) Quality of life evaluation following limb lengthening surgery in patients with achondroplasia. Indian J Orthop 54(Suppl 1):39–46. 10.1007/s43465-020-00127-1 32952908 10.1007/s43465-020-00127-1 PMC7474039

[7] Murton MC, Drane ELA, Goff-Leggett DM, Shediac R, O’Hara J, Irving M et al (2023) Burden and treatment of achondroplasia: a systematic literature review. Adv Ther 40(9):3639–3680. 10.1007/s12325-023-02549-3 37382866 10.1007/s12325-023-02549-3 PMC10427595

[8] Kim SJ, Balce GC, Agashe MV, Song SH, Song HR (2012) Is bilateral lower limb lengthening appropriate for achondroplasia? Midterm analysis of the complications and quality of life. Clin Orthop Relat Res 470(2):616–621. 10.1007/s11999-011-1983-y 21785895 10.1007/s11999-011-1983-y PMC3254769

[9] Laufer A, Rölfing JD, Gosheger G, Toporowski G, Frommer A, Roedl R et al (2022) What are the risks and functional outcomes associated with bilateral humeral lengthening using a monolateral external fixator in patients with achondroplasia? Clin Orthop Relat Res 480(9):1779–1789. 10.1097/CORR.0000000000002209 35471200 10.1097/CORR.0000000000002209 PMC9384902

[10] Ireland PJ, McGill J, Zankl A, Ware RS, Pacey V, Ault J et al (2011) Functional performance in young Australian children with achondroplasia. Dev Med Child Neurol 53(10):944–950. 10.1111/j.1469-8749.2011.04050.x 21838822 10.1111/j.1469-8749.2011.04050.x

[11] Kiss S, Pap K, Vizkelety T, Terebessy T, Balla M, Szoke G (2008) The humerus is the best place for bone lengthening. Int Orthop 32(3):385–388. 10.1007/s00264-007-0327-8 17323094 10.1007/s00264-007-0327-8 PMC2323419

[12] Burghardt RD, Yoshino K, Kashiwagi N, Yoshino S, Bhave A, Paley D et al (2015) Bilateral double level tibial lengthening in dwarfism. J Orthop 12(4):242–247. 10.1016/j.jor.2015.05.006 26566326 10.1016/j.jor.2015.05.006 PMC4601994

[13] Paley D (2021) Extensive limb lengthening for achondroplasia and hypochondroplasia. Children 8(7):540. 10.3390/children8070540 34202538 10.3390/children8070540 PMC8305967

[14] Ko KR, Shim JS, Chung CH, Kim JH (2019) Surgical results of limb lengthening at the femur, tibia, and humerus in patients with achondroplasia. Clin Orthop Surg 11(2):226–232. 10.4055/cios.2019.11.2.226 31156776 10.4055/cios.2019.11.2.226 PMC6526131

[15] Shabtai L, Jauregui JJ, Herzenberg JE, Gesheff MG, Standard SC, McClure PK (2021) Simultaneous bilateral femoral and tibial lengthening in achondroplasia. Children 8(9):749. 10.3390/children8090749 34572181 10.3390/children8090749 PMC8465182

[16] Pawar AY, McCoy TH Jr, Fragomen AT, Rozbruch SR (2013) Does humeral lengthening with a monolateral frame improve function? Clin Orthop Relat Res 471(1):277–283. 10.1007/s11999-012-2543-9 22926491 10.1007/s11999-012-2543-9 PMC3528891

[17] Paley D (2014) Progress in and from limb lengthening. Current progress in orthopedics. Tree Life Media, Mumbai, pp 55–80

[18] Constantinides C, Landorf KB, Munteanu SE (2022) Quality of life, physical functioning, and psychosocial function among patients with achondroplasia: a systematic review. Disabil Rehabil 44(23):6982–6995. 10.1080/09638288.2021.1963853 10.1080/09638288.2021.1963853 34403286

[19] Schiedel F, Rödl R (2012) Lower limb lengthening in patients with disproportionate short stature with achondroplasia: a systematic review of the last 20 years. Disabil Rehabil 34(12):982–987. 10.3109/09638288.2011.631677 22112021 10.3109/09638288.2011.631677

[20] Ginebreda I, Campillo-Recio D, Cárdenas C, Tapiolas J, Rovira P, Isart A (2019) Surgical technique and outcomes for bilateral humeral lengthening for achondroplasia: 26-year experience. Musculoskelet Surg 103(3):257–262. 10.1007/s12306-018-0583-3 30536224 10.1007/s12306-018-0583-3

[21] Beaton DE, Wright JG, Katz JN, Upper Extremity Collaborative Group (2005) Development of the QuickDASH: comparison of three item-reduction approaches. J Bone Joint Surg Am 87(5):1038–1046. 10.2106/JBJS.D.02060 15866967 10.2106/JBJS.D.02060

[22] Daltroy LH, Liang MH, Fossel AH, Goldberg MJ, Pediatric Outcomes Instrument Development Group, Pediatric Orthopaedic Society of North America (1998) The POSNA pediatric musculoskeletal functional health questionnaire: report on reliability, validity, and sensitivity to change. J Pediatr Orthop 18(5):561–571. 10.1097/00004694-199809000-00001 9746401 10.1097/00004694-199809000-00001

[23] Nakano-Matsuoka N, Fukiage K, Harada Y, Kashiwagi N, Futami T (2017) The prevalence of the complications and their associated factors in humeral lengthening for achondroplasia: retrospective study of 54 cases. J Pediatr Orthop B 26(6):519–525. 10.1097/BPB.0000000000000428 28107267 10.1097/BPB.0000000000000428

[24] Savarirayan R, Irving M, Wilcox WR et al (2025) Sustained growth-promoting effects of vosoritide in children with achondroplasia from an ongoing phase 3 extension study. Med 6:100566. 10.1016/j.medj.2024.11.019 39740666 10.1016/j.medj.2024.11.019

[25] Allegri AEM, Bedeschi MF, Bocchi MB, Camurri V, Gonfiantini MV, Leoni C et al (2025) Integrating vosoritide therapy with limb surgery in paediatric patients with achondroplasia: real-life experiences. Orphanet J Rare Dis 20(1):369. 10.1186/s13023-025-03853-7 40676599 10.1186/s13023-025-03853-7 PMC12273278

