# Supplementary material for: Patient perspectives on humeral lengthening in achondroplasia: association between treatment timing and acceptance
Source: Eur J Orthop Surg Traumatol. 2026 Jun 23;36(1):248. doi: 10.1007/s00590-026-04804-z (PMC13290971; doi:10.1007/s00590-026-04804-z)
Supplement: Supplementary file 2 — Supplementary Material 2 [file 590_2026_4804_MOESM2_ESM.docx]

**QUESTIONNAIRE FOR GROUP B**

**1. Activities**

Are you able to perform the following activities independently without assistance?

| **Activity** | **Response** |
| --- | --- |
| Dressing | Yes / No |
| Combing/washing hair | Yes / No |
| Putting on shoes | Yes / No |
| Performing personal hygiene | Yes / No |
| Putting hands into trouser pockets | Yes / No |

Please indicate your opinion regarding the following statements by circling a number from 1 to 5:

**2. Functional Outcome**

I am satisfied with what I am able to do with my upper limbs in daily life.

5 = strongly agree
4 = agree
3 = neither agree nor disagree
2 = disagree
1 = strongly disagree

**3. Aesthetic Component**

I am satisfied with the aesthetic appearance of my upper limbs.

5 = strongly agree
4 = agree
3 = neither agree nor disagree
2 = disagree
1 = strongly disagree

Why did you decide not to undergo humeral lengthening?
(Please explain)

**4. Acceptance of the Procedure**

If I could go back, I would choose to undergo humeral lengthening surgery.

5 = strongly agree
4 = agree
3 = neither agree nor disagree
2 = disagree
1 = strongly disagree

Did you undergo lower-limb lengthening?
Yes / No

If yes, when? ___________________________________

Which segment?
Femur: once / twice
Tibia: once / twice

Considering a score from 1 to 10 (1 = not relevant; 10 = essential for activities of daily living):

• How important was lower-limb lengthening for you? ______

• How important would humeral lengthening have been for you? ______

Comments:

Age at questionnaire completion: __________

Sex: M / F

Thank you for your collaboration.
